# Enhanced XUV harmonics generation from diatomic gases using two orthogonally polarized laser fields

**DOI:** 10.1038/s41598-021-85114-8

**Published:** 2021-03-10

**Authors:** Ganjaboy S. Boltaev, Mazhar Iqbal, Naveed A. Abbasi, Vyacheslav V. Kim, Rashid A. Ganeev, Ali S. Alnaser

**Affiliations:** 1 Department of Physics, American University of Sharjah, PO Box 26666, Sharjah, UAE; 2 Institute of Ion-Plasma and Laser Technologies, Uzbek Academy of Sciences, Tashkent, Uzbekistan 100125; 3 Faculty of Physics, Voronezh State University, Voronezh, 394006 Russia

**Keywords:** Physics, Atomic and molecular physics, Atomic and molecular interactions with photons

## Abstract

Enhanced high repetition rate coherent extreme ultraviolet (XUV) harmonics represent efficient probe of electron dynamics in atoms, molecules and solids. In this work, we used orthogonally-polarized two-color laser field to generate strong even and odd high order harmonics from molecular gas targets. The dynamics of odd and even harmonics from O_2_, and N_2_ gases were investigated by employing single- and two-color laser fields using the fundamental radiation and second harmonic of 1030 nm, 37 fs, 50 kHz pulses. The relative efficiencies of harmonics were analyzed as a function of the thickness of the barium borate crystal used for second harmonic generation. Defocusing-assisted phase matching conditions were achieved in N_2_ gas for different groups of XUV harmonics.

The pursuit for table top intense coherent sources in different ranges of extreme ultraviolet (XUV) drives the continuous investigations of high-order harmonic generation (HHG) during the interactions of ultrashort infrared femtosecond pulses with atoms, molecules, laser plasma, and solid targets^1–6^. HHG from gas targets possesses great potential for probing and controlling electron dynamics with attosecond time resolution, where enhanced XUV harmonics are required^7^. Attosecond tomography is based on the exploration of the HHG spectra generated from atomic and molecular targets and was demonstrated in^8^. However, a common drawback in gas HHG is the low conversion efficiency and consequently the low photon flux of the high-order harmonics. To overcome the hurdle of low photon flux of the XUV harmonics, the use of successive sources allowing the enhancement of photon flux has been proposed^9^. Particularly, higher conversion efficiency of XUV harmonics has been achieved by employing two-color field of gases using orthogonally-polarized two-color laser pulses^10,11^, where the conversion efficiency of harmonics can be increased up to ~ 10^−4^ when two-color field is applied.

The low-conversion efficiency of XUV harmonics is one of the challenges faced in generating and employing attosecond pulses with high energy. Due to its capability in increasing the HHG conversion efficiency, the approach of employing two-color laser field has been the subject of theoretical and experimental investigations^12–14^. The theoretical concepts of efficient generation of XUV harmonics using bichromatic laser pulses were analyzed earlier^15^. It was shown that the enhancement factor of harmonics in gases relies on the intensity ratio of the fundamental pulse and its second harmonic (SH). Moreover, the polarization of the generated harmonics can be determined by the polarization state of either the main or SH pulse. Linearly-polarized radiation can be obtained with linearly polarized bichromatic laser pulses, whereas circularly polarized radiation can be generated with bichromatic circularly polarized pulses with counter-rotating coplanar components^15^. Additionally, amended conditions for HHG through plasma-induced defocusing-assisted phase-matching (DAPM) of the driving pulses in gases can be also considered for enhancing the XUV harmonics yields^16^.

Phase mismatch of the interacting waves can restrict the conversion efficiency of harmonics^17,18^. The generation of bright harmonics in the range above 20 eV using two-color field was reported in^19^, where formation of highly-efficient source of femtosecond XUV pulses at 50 kHz repetition rate was implemented under tightly focusing of ultraviolet SH (2 $\omega$; at the wavelength of λ = 390 nm) into Kr gas jet. The enhancement arises from both the wavelength scaling of the atomic dipole and improved spatio-temporal phase matching. XUV harmonics with high repetition rate enable the application of HHG spectroscopy to study complex molecular systems^17^, and probe the asymmetries in pre-oriented polar molecules^20^. Correspondingly, the application of two-color field to cover this spectral range with strong harmonics can be utilized in analyzing the dynamics of electrons in various quantum materials^21^.

In this work, we investigate HHG in gases of similar ionization potentials using single-color pulses ($\omega$) and orthogonally-polarized two-color pulses ($\omega$ + 2$\omega$) at 50 kHz repetition rate. We demonstrate the enhancement of harmonics in different spectral ranges (between 15 and 50 eV) from N_2_ and O_2_ gases using nonlinear crystals with different thicknesses for the generation of the second harmonic. The extension of XUV harmonic cutoff is demonstrated. Further, we exploit two-color field and DAPM conditions to control the relative yields of even and odd harmonics by changing the energy of driving beam and the geometrical parameters of the focused pulses.

## Results

### HHG in molecular gases by controlling the energy of driving pulses

**Figure 1:**
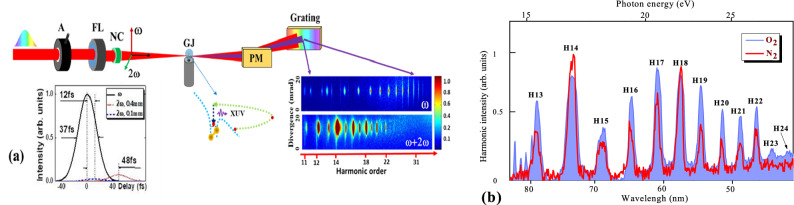
(**a**) Experimental arrangements for the generation of coherent XUV radiation using high-repetition rate near-infrared Yb fiber laser source. The laser pulses were propagated through the variable aperture iris (A) and focused by focusing lens (FL) onto the gas jet (GJ). The nonlinear crystal (NC) was inserted on the path of laser beam inside the vacuum chamber. The generated harmonics were analyzed using an XUV spectrometer with gold-coated plane mirror (PM) and flat field grating. The harmonic spectra were collected by CCD camera. The graph beneath optical axis shows the temporal map of separated pulses after propagation through different nonlinear crystals (*λ*_1_ = 1030 nm, black curve; *λ*_2_ = 515 nm, blue dashed curve in the case of 0.1 mm thick BBO and red dotted curve in the case of 0.4 mm thick BBO). The scheme beneath the gas jet depicts the three-step mechanism of HHG. Inset: Raw images of the odd harmonics spectra generated in oxygen in the case of single-color field (upper panel) and odd and even harmonics in the case of two-color field using 0.4 mm thick crystal (bottom panel). (**b**) Typical lineouts of harmonic spectra generated in N_2_ and O_2_ using orthogonally-polarized two-color ($\omega$ + 2 $\omega$) field.

**Figure 2:**
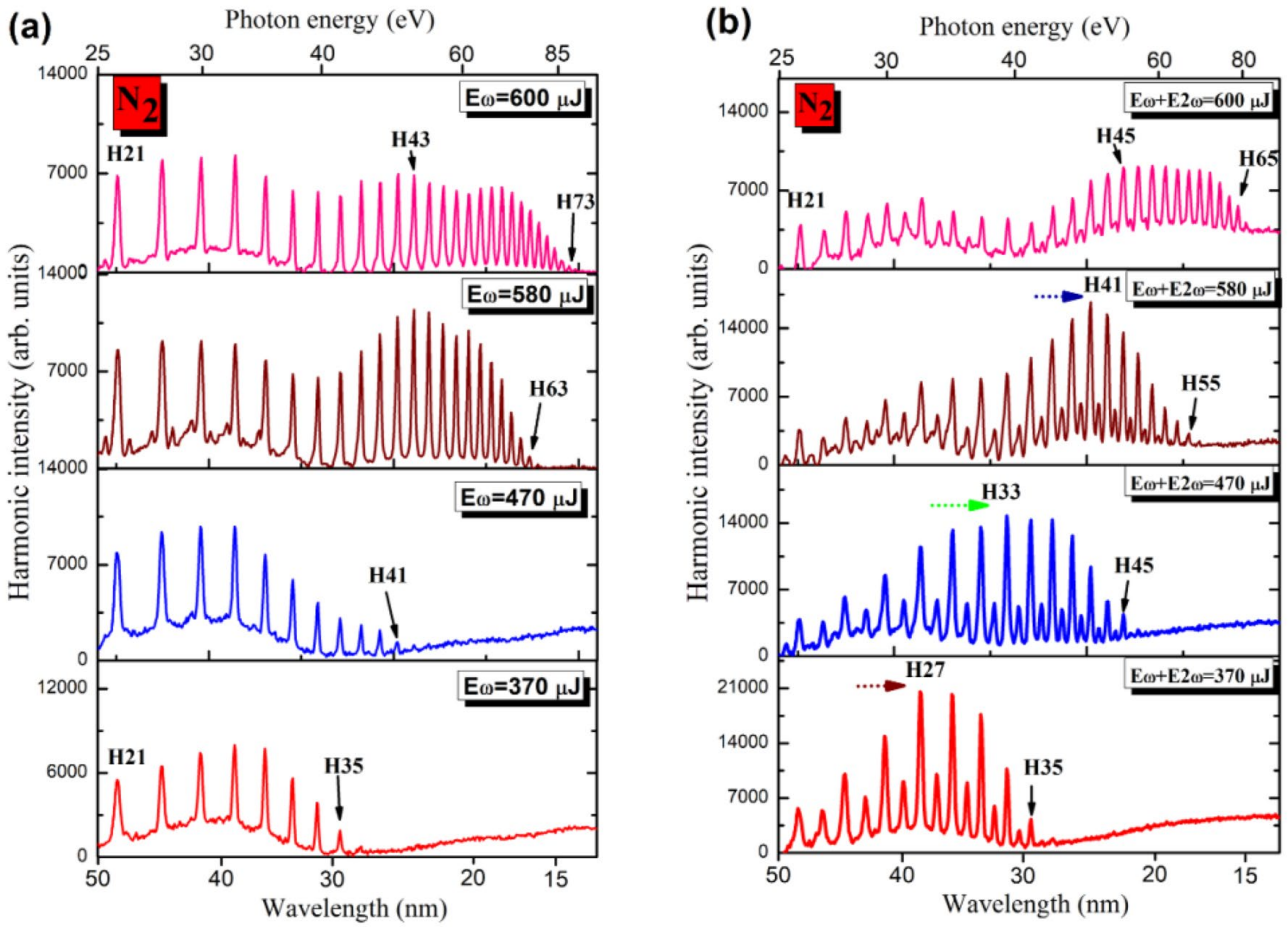
Spectra of XUV harmonics generated in N_2_ using (**a**) Single-color field ($\omega$) at the wavelength of 1030 nm; and using (**b**) Two-color field ($\omega$ + 2 $\omega$) using orthogonally-polarized 1030 and 515 nm pulses. The SH pulses with 2.8% conversion efficiency were generated in 0.1-mm thick crystal. Laser intensity in the gas jet was 2.1 × 10^14^ W/cm^2^, and gas pressure was 200 mbar. The dotted arrows (red, green, and blue) show the shift of the position of DAPM enhanced harmonics at different energies of two-color field.

Detailed description of the experimental setup is provided in the methods section (Fig. 1). Figure 2a shows the spectral distribution of harmonics generated in N_2_ gas using 1030 nm, 37 fs driving pulses at different pulse energies. To compare the energy dependence in the case of two-color field, we used 0.1 mm thick barium borate (BBO) crystal. In the case of single-color field, increasing the energy of the driving pulses led to extension of the cutoff energy of harmonics. At the lowest used energy (370 µJ), the highest generated order was H35 (42.1 eV photon energy). Further increase in the energy of the driving pulses led to the growth of the observed order of harmonics up to H73 (88 eV) at *E*_ω_ = 600 µJ. The relation between the HHG cutoff energy and the intensity (or energy) of fundamental wave was described in^22,23^, which predicts the (*E*_*cutoff*_* ∞ I*_*ω*_) dependence. Similar variation in the cutoff energy was observed in the case of two-color field (Fig. 2b).

Controlling the energy of driving pulse with an iris can also lead to variations in the defocusing-assisted phase matching, which is shown in the variable spectra of harmonics generated in nitrogen in the case of two-color laser pulses. The ratio of the driving pulse and its second harmonic energies was kept at 30:1. The enhancement factors for 27th and 33rd harmonics generated in N_2_ by two-color laser field were equal to 1.7 × and 2 × when compared to the single color case at the same total driving energy.

HHG in N_2_ gas was studied using radiation of classical Ti: sapphire laser system at 1 kHz repetition rate driving pulses^24^. The cutoff energy of XUV harmonics generated in N_2_ gas was recorded up to 57th order at high intensity (~ 9.0 × 10^14^ W cm^−2^). Optimization of HHG in noble gases by changing the beam diameter using single color 800 nm driving pulses was demonstrated previously^25^. In our work, we exploit the influence of variable aperture on the HHG spectra from diatomic molecules when single-color field and two-color field configurations are employed. The application of two-color laser pulses led to the enhancement of some groups of harmonics when the largest aperture was used to allow highest energy of driving pulses (see H45–H51, Fig. 2b). We were able to control the range of enhanced harmonics by decreasing the diameter of the iris such that the positions of enhanced harmonics (H41, H33, and H27, respectively) were shifted towards longer wavelength XUV region compared to the upper panel case. The arrows in the three bottom panels of Fig. 2b show the positions of enhanced harmonics in the case of two-color field at 8 mm, 12 mm, and 16 mm diameters of iris.

**Figure 3:**
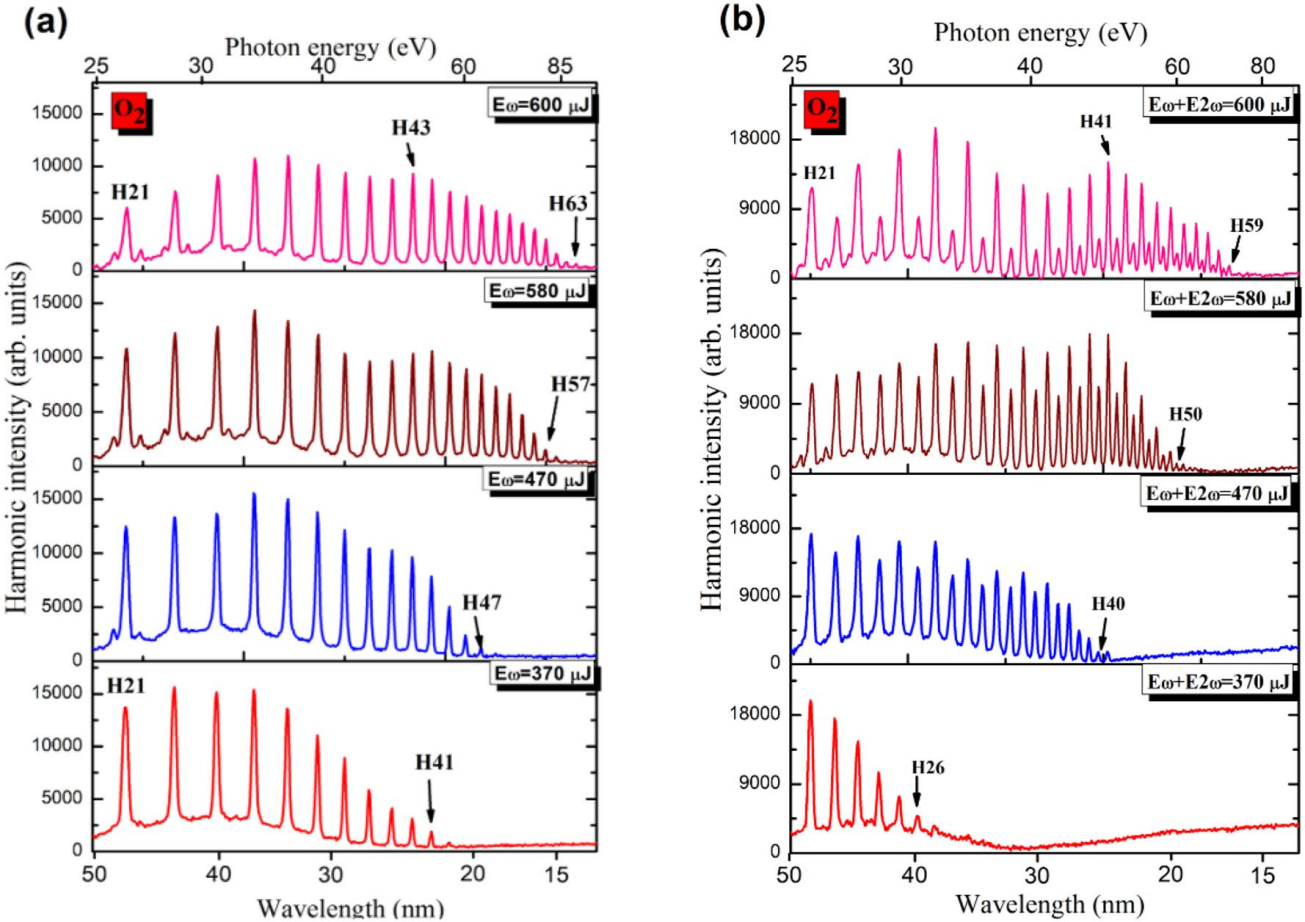
Spectra of harmonics generated in O_2_ gas using (**a**) single-color field and (**b**) two-color field. In the cease of two-color field, the SH with CE = 2.8% was generated in 0.1-mm thick crystal.

By comparing the single-color field-induced with the two-color field-induced spectra of harmonics at the lowest energy applied (bottom panels of Figs. 2a,b), a similarity in the harmonic cutoffs in the two cases was observed. The presence of even harmonics, though weaker than the odd ones, is noticed up to the highest energy of generated harmonics (40 eV, bottom panel of Fig. 2b), despite their weak presence at this spectral range. This is due to the quadratic dependence (*E*_*cutoff*_ ∞ *λ*^2^)^22^ on the wavelength of the driving pulses, especially with separate participation of the relatively strong 1030 nm field and relatively weak 515 nm field in the HHG, where small conversion efficiency (CE) of SH takes place at smaller energy of fundamental radiation (Fig. 2b). A possible explanation of this observation could be as follows: At certain setting of the iris diameter (~ 8 mm), the involvement of DAPM may create the conditions for harmonics generation in the spectral range where they are not expected to appear at two-color field of the gas target. The increase of the laser beam diameter led to separating the harmonic cutoffs for odd and even orders while shifting the maximally enhanced harmonic towards the high photon energy range. Note that DAPM also affects the odd-orders harmonic at the low energy part of the driving pulses. Similar experiments on O_2_ gas showed that cutoffs for odd and even harmonics were almost the same (Fig. 3b). Figure 3 shows spectra of harmonics generated in O_2_ gas by using single-color and two-color field configurations at the experimental conditions similar to the N_2_ case.

As mentioned, the similarity in harmonic cutoffs for odd and even harmonics and the absence of the group of enhanced harmonics in O_2_ (Fig. 3b) notably differ in those spectra when compared to the two-color field induced HHG in N_2_ (Fig. 2b). The observed extension of XUV harmonics in O_2_ (ionization potential *I*_*p*_ = 12.07 eV) can be explained by the ionization suppression of O_2_ at the high intensity of driving pulse, which was also observed in^23^. At intensities ($I_{\upomega }$ = 1.4 × 10^14^ W cm^−2^ and $I_{\upomega }$ = 1.3 × 10^13^ W cm^−2^) of the two driving pulses we obtained even and odd harmonics with the same outputs (Fig. 3b, ${\text{E}}_{\upomega }$ + ${\text{E}}_{{{2}\upomega }}$ = 470 $\upmu$ J).

**Figure 4:**
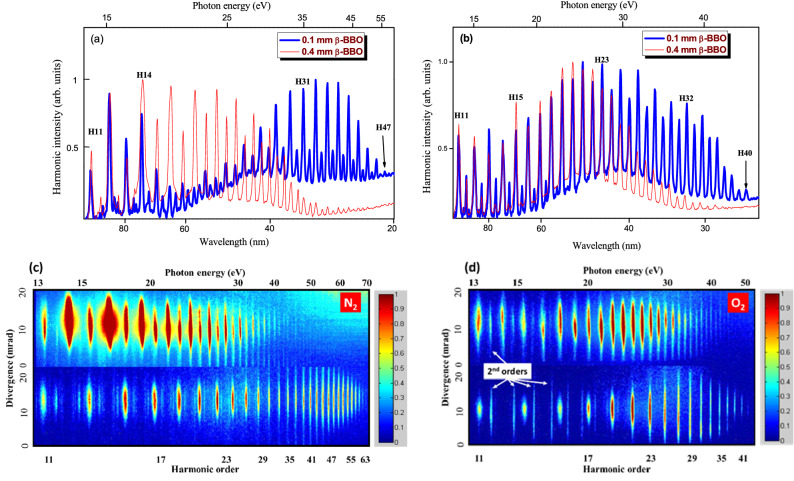
Normalized intensities of harmonics generated during two-color field of (**a**) nitrogen and (**b**) oxygen using BBOs of different thickness (0.1 mm: thick blue curves; 0.4 mm: thin red curves). The harmonics were obtained at E_ω_ + E_2ω_ = 470 µJ. Raw images of harmonics generated in (**c**) N_2_ and (**d**) O_2_ using 1030 nm pulses (bottom panels in (**c**) and (**d**)) and (1030 nm + 515 nm pulses) (upper panels in (**c**) and (**d**)). The total energy of driving pulses was equal to 520 and 470 **µJ for the experiments with N_2_ and O_2_ gases, respectively. 2nd order spectral lines are marked with white arrows in Fig. 4d. These lines correspond to the second-order emission of strong higher order harmonics from the 23rd to the 35th orders.

Figure 4 presents the comparative spectra of XUV harmonics generated in N_2_ and O_2_ using the two-color laser pulses when using nonlinear crystals of different thickness. The total energy of two driving pulses was equal to 470 $\upmu$ J. The delays between two pulses after propagation through the 0.1 and 0.4 mm thick crystals were calculated to be 12 and 48 fs, respectively. From the observed harmonics spectra we can see the variation in the range of enhanced HH when nonlinear crystals of different thicknesses are used. For enhancement of intensities of HHGs in relatively low-photon energy range we used 0.4 mm thick nonlinear crystal with 8% CE. In this case the delay between two orthogonally polarized pulses was equal to 48 fs (see inset in Fig. 1a). In the meantime, the two-color field using 0.1 mm thick $\beta$-BBO allowed extending the range of enhanced harmonics up to 40th order in O_2_ gas (Fig. 3b). By analyzing the raw images of XUV harmonics (Figs. 4c,d) generated from the two diatomic gases, the enhancement ratio for the 11th order harmonics in the two-color laser fields, compared to the single-color field, were equal to 3 × and 3.6 × for N_2_ and O_2_, respectively.

Moreover, the installation of gold-coated plane mirror in the XUV spectrometer allowed analyzing the divergence of harmonics in different spectral ranges. One can see the distinction in the divergences of the harmonics generated in the cases of single- and two-color field.

### Divergence and phase matching of harmonics

Figure 4c presents the raw images of HHG spectra from nitrogen obtained using 16 mm iris with total energy of 520 $\upmu$ J, while Fig. 4d was obtained during HHG from oxygen in the case of 14 mm iris (*E*_ω_ + *E*_2ω_ = 470 $\upmu$ J). In the first case (N_2_), even harmonics were exceptionally strong in the region of 14–21 eV, while in the second case (O_2_), a notable enhancement of shorter-wavelength even harmonics was achieved in the region of 22–29 eV. The larger divergence of highest orders of harmonics in the case of single-color laser pulses points out to the involvement of long trajectories of accelerated electrons, alongside the short trajectories in harmonics generation^26^. The divergence of XUV harmonics generated in isotropic media, from the short and long trajectory, is given by^27–29^:

1 $$ \theta_{S,L} = \frac{{\lambda_{q} }}{{\pi D_{q} }}\sqrt {1 + 4\alpha_{S,L}^{2} I_{\lambda }^{2} \frac{{D_{q}^{4} }}{{D_{\lambda }^{4} }}} $$

where $\theta_{S,L}$, $\alpha_{S,L}^{2}$ are divergence and divergence coefficient originated from the short (S) and long (L) trajectory of electron after tunneling ionization from atom at the peak of driving laser field; $\lambda_{q}$ , D_q_ is the wavelength and beam diameter of the qth order harmonic; and $I_{\lambda }$, and $D_{\lambda }$, are intensity and beam waist of the driving laser pulses. Analysing the divergence of XUV harmonics has been routinely employed to infer the contributions of the active electron’s short- and long trajectories in the HHG spectra from atomic targets^27–29^. The variation of divergence of XUV harmonics (Figs. 4c,d) generated from the two diatomic gases can be used to infer the contribution of short and long trajectories in producing those harmonics. Although contributions from either trajectory cannot be excluded for any harmonic, the variation of the divergence at a specific harmonic order can be used to infer the relative contribution of that trajectory. Identifying those contributions can be then used to select certain trajectory by controlling the phase between the two-color field driving those harmonics.

**Figure 5:**
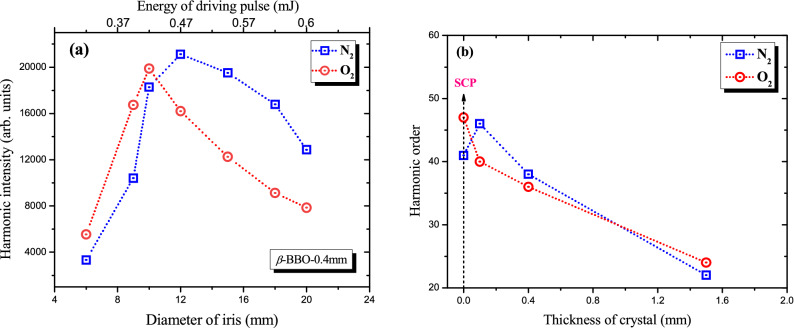
(**a**) Variations of 16th harmonic intensity in the case of nitrogen (empty blue squares), and oxygen (empty red circles), as a function of the iris diameter. The ratio of energy between fundamental (1030 nm) and SH (515 nm) pulses was kept at 10:1. (**b**) Dependence of the highest order harmonic on the thickness of nonlinear crystal in the case of two-color field of HHG in N_2_ (empty blue squares) and O_2_ (empty red circles) gases: Black dashed arrow shows the number of harmonic order for single-color pulse.

Figure 5a shows the yield variations of the 16th harmonic generated from N_2_ and O_2_ using two-color laser field. One can see that at 10 mm (for O_2_) or 12 mm ( for N_2_) diameters of iris, a significant difference in harmonic yield was observed compared to smaller and higher sizes of aperture, which points out to optimization in the phase matching conditions. In this dependence firstly the harmonics intensity was increased by increasing total energy of driving pulses up to ~ 0.47 mJ, and then further increase in the total energy of the driving pulses led to monotonic decrease in the intensity of 16th harmonics generated in N_2_ and O_2_ gases. The decrease of harmonic yields, when driving pulses of high intensity are used, is attributed to the ionization of gases during the propagation of laser pulses. In this case relation of neutral and ionized gas dispersion can lead to phase-mismatching between the interacting waves. Figure 5a shows that it is possible to control intensity of even order harmonics by tuning the spatial and energetic parameters of driving pulses at the wavelengths of 1030 nm and 515 nm. Similar behavior of the HHG intensity was observed by changing the energy of single-color laser pulses from noble gases^22^. By controlling the shape of driving femtosecond pulses one can enhance the CE of HHG and achieve strong temporal confinement of harmonics as was also predicted earlier^30,31^. This kind of beam shape controlling can also allow for finding the laser beam parameters that would lead to generating stronger XUV harmonics. The variation of beam sizes by using variable-diameter aperture can optimize maximal harmonic yield due to the variation of focal geometry and ionization rate of diatomic gases.

Figure 5b shows the dependence of highest order harmonic on the thickness of nonlinear crystals ($\beta$-BBO, type I) at the intensities ($I_{\upomega }$ = 1.4 × 10^14^ W cm^−2^ and $I_{\upomega }$ = 1.3 × 10^13^ W cm^−2^) of fundamental radiation and it’s SH, respectively. As shown in the figure, a change in the highest order of harmonics in N_2_ and O_2_ gases is observed. The abnormal harmonic order (41th) was observed in N_2_ gas. Using two-color field with 0.1 mm thick BBO led to increase in the highest order (45th) of harmonic compared to single-color field at the same intensity of fundamental driving pulse. Due to the wavelength scaling of HHG (*E*_*cutoff*_* ∞ λ*^2^)^22^ the cutoff energy of harmonics should decrease as presented in O_2_ gases (empty red circles). In the case of HHG in N_2_ gas using two-color pulses, DAPM was realized for simultaneous interaction of two orthogonally-polarized laser pulses with N_2_ gas. This is also supported by the results shown in Fig. 2a,b at the energy (*E*_ω_ = 470 $\mu$ J and *E*_ω_ + *E*_2ω_ = 470 $\mu$ J) of the driving laser pulses, respectively. We present these dependences for all three nonlinear crystals with 0.1, 0.4, and 1.5 mm thicknesses at the same energy of driving laser pulses.

## Discussion

The interest in generating coherent XUV radiation drives the search for methods that would lead to higher order harmonics enhancement, particularly using two-color field, which allows increasing the tunneling ionization rate in the presence of a mixed electromagnetic fields. In the past three decades, most of the efforts were performed on noble (atomic) gases ^14,32,33^. Meanwhile, the reports on HHG results from molecular gases have been comparatively rare. Theoretical studies on HHG from molecules were carried out demonstrating increase in the conversion efficiency of high harmonic generation^2^. Due to the presence of the additional degrees of freedoms such as the alignment and vibration in molecules the possibility to control the phase of the nonlinear polarization of the medium and of meeting the phase-matching condition were predicted. Additionally, maximizing and minimizing certain harmonics were demonstrated by varying the alignment of CS_2_ and N_2_ molecules with respect to the laser field^34^. Applying the two-color field approach on N_2_ and O_2,_ along with the other ubiquitous macroscopic processes that would be tuned through DAPM, can make those two diatomic gases efficient emitters of harmonics. Moreover, the polarization state of the two driving pulses is important factor in any two-color field study. The influence of polarization state of two different pulses has already been analyzed in noble gases^10^. According to the Ammosov-Delone-Krainov theory^35^, the total ionization rate in the case of parallel polarization of two driving field is about 90%, which is much larger than that in the case of orthogonal polarization (30%). The high ionization rate in the parallel polarized fields makes it difficult to maintain the phase matching conditions because, under such conditions, the driving field experiences severe self-phase modulation in the ionized medium and decrease of HHG conversion efficiency.

The propagation of two-color field can be expressed by following Eq.^10^:

2 $$ E\left( t \right) = {\text{exp}}\left[ { - 2ln2\left( {\frac{t}{\tau }} \right)^{2} } \right] \times \left[ {E_{1} \cos \left( {\omega t} \right){\hat{\mathbf{x}}} + E_{2} \cos \left( {2\omega t + \phi } \right){\hat{\mathbf{y}}}} \right], $$

where $E_{1}$ and $E_{2}$ are the amplitudes of the strong fundamental and weak second harmonic fields, $\tau$ and $\omega$ are the pulse duration and frequency of the fundamental field, and $\phi$ is a relative phase of the second harmonic field with respect to the phase of fundamental field at the output of the nonlinear crystals. In our case, we used good quality BBO crystal, which allowed ignoring the phase shift between fundamental driving field and its SH. Equation 2 (with zero phase shift between fundamental and its second harmonics) allows explaining the role of ellipticity of the driving two-orthogonally polarized laser field on the XUV harmonics output and cutoff. As was described in Ref.10, the Lissajous diagram for orthogonally polarized electric fields correspond to variation in the relative field strength of the fundamental and SH laser pulses. The recollision of the excited electron with its parent ion occur in the linear component of the two-orthogonally polarized fields, taking into account the intensity ratios of the fundamental and SH laser pulses. The different intensity ratios ($I_{\omega }$/${\text{I}}_{2\omega }$ ~ 40, 20, 10) between two-color laser fields generated in 0.1 mm, 0.4 mm, and 1.5 mm thick-BBO crystals led to the enhancement of XUV harmonics in different ranges of HHG with photon energies from 15 to 50 eV; and showed dependence of cutoff energy of XUV harmonics generated in diatomic gases on the thicknesses of the nonlinear crystals (Fig. 5b). Moreover, evidence for the spatial overlap of the driving beams is the symmetrical distribution of the odd and even harmonics orders with respect to the axis of dispersion of XUV radiation (Fig. 1a, see the raw image of HHG spectrum in the case of two-color field). The raw images of harmonic spectra presented in Figs. 4c,d also show the almost perfect spatial overlap of two laser beams in the area of interaction resulting in almost equal groups of odd and even harmonics positioned on the same axis of dispersion of the flat field grating. Notice that placement of BBO on the pass of focused radiation inside vacuum chamber diminished the walk-off of two pulses during propagation of beams through the optical elements. Defocusing assisted phase matching (DAPM) of interacting waves was analyzed in^16^ by using 800 nm driving pulses. This process was experimentally and theoretically demonstrated for different lengths (0.8, 1.2 and 7 mm) of gas cells. It was also demonstrated that at the high-intensity of driving pulses, the radial gradient of the electron density can cause defocusing of the propagating laser beam through gas jets with variable lengths. Varying the iris aperture allows for tuning intensity of the driving pulses and reaching defocusing assisted phase matching (DAPM) conditions, which helps in extending the harmonic cutoff^36^. DAPM can be achieved with a tightly focused beam and highly ionized short gas cell by tuning the intensity gradient of driving pulse along the propagation direction and position of the gas target. In our case, the N_2_ /O_2_ gas jet was installed at the focal plane of the focusing lens to reach high intensity for the driving two-color laser fields. The focusing conditions provided spot size of 50 μm radius. The corresponding confocal parameter of the focused diffraction-limited beam was *b*∼15.2 mm. Due to the fact that $l < b$ (with $l$ being the jet width), the intensity gradient of the driving pulses enabled the realization of DAPM and its influence on the generated HHG spectra in both molecular targets. The enhanced group of harmonics gradually tuned towards the higher orders (i.e., shorter-wavelength region) with the growth of the energy of the driving pulses. This shift of the spectral range of enhanced harmonics can be attributed to the dependence of the coherence length ($L_{coh} \approx 10^{18} /N_{e} \times q$, where *N*_*e*_ is the concentration of free electrons and *q* is the harmonic order) of *qth* harmonic on the concentration of free electrons appearing in gas during propagation of strong driving pulses (see Fig. 2b). As shown in Fig. 2a, the almost two-fold decrease of laser pulse energy (from 600 to 370 µJ) led to almost two-fold decrease of the cutoff energy and harmonic order of generated coherent emission (H73 and H37, Fig. 2a). The variation of the spatial characteristics of the driving beam after propagating through the partially-opened iris would result in changing the beam waist diameter of the focused radiation, which would influence the value of the calculated intensity of the driving pulses. Additionally, the transmission through the iris did not linearly scale with the diameter of its aperture. For instance, while we used 8 mm and 20 mm diameters of iris, through which the driving pulse propagated, the transmitted energies from those diameters were equal to 370 µJ and 600 µJ, respectively. One can see that although the ratio between diameters was equal to 2.5, the ratio between energies of pulses was equal to ~ 1.6, which is expected in the case of Gaussian beams. Furthermore, influence of macroscopic processes can be responsible for the lack of direct correlation between the used intensity of laser radiation and the measured value of the cut-off energy.

The appearance of even harmonics spectra was previously reported and analyzed in^37,38^. It was demonstrated that the addition of weak second harmonic field significantly enhances the short path contribution while eliminating other electron paths, resulting in a clean high-harmonic spectrum through suitable control of the relative phase between the fundamental and second harmonic waves. High order harmonics spectrum generated by single-color laser pulses contains contributions from different electron paths including the long paths, which are more sensitive to the laser intensity and more prone to spectral broadening and shifting. Compared to the spectra generated by two-color laser field, where contributions from shorter paths become more significant, HHG spectra form single-color field are more complex^37^. In our two-color experiments, each of the laser field components can separately generate its own odd-order harmonics, which in the case of single-color laser pulses corresponds to odd ones ((2n + 1)) and in the case of second harmonic field correspond to even (2(2n + 1)) orders. For the (2n + 1)th orders, the fundamental field is the essential contributor, and the presence of the second field enhances the signal from the short paths. The 2(2n + 1)th orders can be generated by the second harmonic field alone. The continuous increase of the intensity of low-order harmonics indicates that the second field is the main contributor to these orders. Once the fundamental and second harmonic pulses overlap with each other in the gas medium, the parametric process can cause the appearance of the sum and different harmonics due to mixing of the waves in the nonlinear medium, which leads to the appearance of the 4(n + 1)th orders (H8, H12, H16, H20, etc.) when both fields overlap. Those orders can be generated significantly by adding a weak fundamental field to strong second harmonic field or by adding a weak second harmonic field to strong fundamental field. These orders disappear when one of the two pulses is absent or there is very weak temporal or spatial overlap between these two pulses, which was observed in our studies.

The spectra obtained at higher intensities present interesting features of phase matching. Below we qualitatively address and characterize the spectra obtained at higher intensities, which show features of phase matching. Among the different methods of harmonic enhancement, the formation of the phase matching conditions between the driving and generated waves during HHG should be considered^38,39^. The conditions of harmonics enhancement can be maintained along the whole medium length (so-called coherence length, *L*_coh_) until the phase difference Δ*φ* between converting and converted waves becomes equal to *π*. The coherence length of the *H*_pm_ harmonic is determined as *L*_coh_ ≈ 4*π*^2^*m*_*e*_*ε*_0_*c*^2^*/H*_pm_*N*_*e*_e^2^*λ,* where *λ* is the wavelength of the driving radiation; *m*_*e*_, and *e* are the mass, and the charge of an electron; *c* is the velocity of light, and *ε*_0_ is the permittivity of vacuum^40^. *H*_pm_ is the maximally enhanced harmonic in the spectral range suitable for phase matching and *N*_*e*_ is the electron density in cm^−3^. Free electrons can appear at the intensities exceeding the first and second ionization potentials of harmonic emitters. The variation of free electron concentration in gases and plasmas allows the tuning of the group of enhanced harmonics, like in the cases shown in Figs. 2 and 4.

The phase mismatch induced by gas dispersion for the *H*_pm_ harmonic is defined by the relation *Δk*_disp_ = *H*_pm_ *N*_*e*_e^2^*λ/*4*πm*_*e*_*ε*_0_*c*^2^*.* One can deduce the relation (*H*_pm_ ∞ *λ*^−1^) between the maximally enhanced harmonic in the phase matching range and the laser wavelength. Correspondingly, the use of twice shorter wavelength leads to the two-fold increase of the order of maximally enhanced group of harmonic. Thus the observation of phase matching at the conditions of two-color field is possible only in the case when the gas medium allows generation of twice longer cut-off than that required for observation of the phase matching using single-color field. Our studies showed that the influence of second harmonic was sufficient to strongly enhance and modify the whole harmonic spectrum compared to single-color field.

Finally we present the divergence dependence on the harmonic order as a demonstration of the spatial overlap of the two driving beams. The symmetrical distribution of the XUV harmonics in the raw images allowed concluding about the proper spatial overlap of two driving beams during propagation through the gas jet maintained at the focal plane of focusing lens. The higher-order phase matched harmonics showed higher divergence in the case of single-color field (bottom panels of Figs. 4c,d). Meanwhile, in the case of two-color field, the divergence decreased with the increase of harmonic order as one can expect. The presence of phase matching features in the case of 1030 nm beam points out to increase in the role of macroscopic processes compared to the single-atom (microscopic) effects, which may include, alongside the involvement of long trajectories, the increase of the harmonics divergence. On the other hand, in the case of two-color field, short trajectories are enhanced, which leads to narrowing the divergence for highest orders and the appearance of harmonics dominantly on the axis of laser beams propagation. Although contributions from either trajectory cannot be excluded for any harmonic, the variation of the divergence at a specific harmonic order can be used to infer the relative contribution of that trajectory. Identifying those contributions can be then used to select certain trajectory by controlling the relative phase between the two-color fields driving those harmonics^41^.

## Conclusion

We have demonstrated the enhancement of XUV harmonics intensity generated in diatomic gases using orthogonally-polarized two-color laser pulses (fundamental radiation and SH of 1030 nm, 37 fs, 50 kHz pulses). It has been shown that changing the driving pulse energy and time-delay influences the yield of XUV harmonics generated in the interactions with N_2_ and O_2_ molecular targets. The different time-delay shifts (12 fs, 48 fs, and 133 fs) with different intensity ratios ($I_{\omega }$/${\text{I}}_{2\omega }$ ~ 40, 20, 10) between the two-colour laser fields generated in 0.1 mm, 0.4 mm, and 1.5 mm thick-BBO crystals led to the enhancement of XUV harmonics in a wide range of HHG with photon energies from 15 to 50 eV. We presented the dependence of the cutoff energy of XUV harmonics generated in diatomic gases on the thicknesses of the nonlinear crystals. We have also demonstrated the possibility of controlling the intensity of even harmonics by showing the correlation between the yield of the16th order harmonic and the size of the iris that controls the energy of the driving two-color laser pulses. Through the DAPM approach with two-color laser field, we demonstrate enhancement of the 27th and the 33rd harmonics generated in N_2_. Furthermore, enhancement factors of 3 × and 3.6 × of the 11th order were achieved in two-color laser fields compared to the single-color field, for N_2_ and O_2_, respectively.

## Methods

### Experimental arrangements for XUV harmonics generation

The experimental setup is shown in Fig. 1a. We used active fiber laser system, which provides a 37 fs pulses at 50 kHz repetition rate with an energy of 0.6 mJ at central wavelength of λ_1_ = 1030 nm ($\omega$). The driving pulses with Gaussian-like spatial distribution were focused into a gas jet (GJ) using 400 mm focal length focusing lens (FL). To control the position of gas jet with respect to the focal plane of focusing lens we used 3-D motorized translating stage (SmartAct Positioning Systems). The position of the focal plane was determined using CCD camera-based beam profiler (Thorlabs). The position of gas jets was kept at the focal plane during the studies of XUV harmonics generated in diatomic N_2_ and O_2_ gases.

The radius of the beam waist at the focus of the driving pulse was equal to 50 $\mu$ m. The maximal intensity of the fundamental beam inside the gas jet at these focusing conditions was ~ 2.1 × 10^14^ W cm^−2^ for a 20 mm diameter of the iris (A) placed in front of focusing lens, when almost all energy of laser beam propagated through this iris. The variation of the diameter of this iris was used to control the energy of driving pulses and the geometrical parameters of focused beam. The energy of driving pulses was varied between 0.25 and 0.6 mJ when the diameter of iris varied from 6 to 20 mm. The variation of the beam’s diameter allowed modifying the defocusing assisted phase matching conditions in the laser-gas interaction area. Beta-barium borate ($\beta$ -BBO, type I) crystals with different thicknesses were used for second harmonic generation (2 $\omega$; at the wavelength of *λ*_2_ = 515 nm).

These nonlinear crystals (NC) were inserted inside the vacuum chamber on the path of the focused beam at a distance of 200 mm from the focusing lens. The SH conversion efficiencies in the case of 0.1, 0.4, and 1.5 mm thick BBO crystals were measured to be 2.8, 8 and 10%, respectively. The efficiency for 1.5 mm crystal was almost the same as for the 0.4 mm crystal, since being inserted on the path of the focused beam the thicker crystal did not notably improve CE due to the growing influence of the group velocity dispersion in the thick nonlinear crystal.

The inset in Fig. 1a shows the temporal overlap of two pulses (ω and 2ω) after leaving the nonlinear crystal and reaching the gas area. The temporal dependence on the intensity variations of these laser fields quantitatively shows the temporal delay between fundamental and SH waves propagating with different group velocities through the 0.1 and 0.4 mm thick BBOs. The ratio of the delays between two pulses is 4 (12 and 48 fs, see below), which corresponds to the ratio between the thicknesses of these crystals. One can see that in both cases the ω and 2ω pulses sufficiently overlap in the gas area. The inset also depicts the relative intensities of waves (1:0.08, 1:0:0.028) and the difference in the overlaps when two thin crystals (0.4 and 0.1 mm) at the 48 and 12 fs delays respectively. Additionally, the raw images of XUV harmonics presented in the same picture demonstrate a proof of the sufficient temporal overlap of two pulses in the gas area resulting in the appearance of all even harmonics (H12, H14, H16, H18, H20, H22, and so on), contrary to the case when, for insufficient overlap of ω and 2ω waves, one can generate only 2(2n + 1) even harmonics (H10, H14, H18, H22, and so on). These raw images also show the almost perfect spatial overlap of two laser beams in the area of interaction with gas atoms, which resulted in the appearance of almost equal groups of odd and even harmonics positioned on the same axis of the dispersion of flat field grating. Notice that the installation of BBO inside the vacuum chamber on the path of focused radiation decreased the walk-off of two beams during their propagation to the gas target.

The pressure in the gas chamber was ~ 3.5 × 10^–3^ mbar during the experiments, while the XUV spectrometer chamber was maintained at ~ 3 × 10^–5^ mbar. The interaction length was estimated to be equal to the inner diameter (~ 1 mm) of the cylindrical gas jet. The position of GJ was kept at the focal plane (z = 0) of the focusing lens. The harmonics were analyzed using the XUV spectrometer consisting of a flat field grating and micro-channel plate detector coupled to a phosphor screen. The raw images of the harmonic spectra were collected using a charge-coupled device camera (inset in Fig. 1a). Notice that these images were obtained at the saturated regime of collection for better viewing of harmonic distribution by eye, while the line-outs presented in the following figures were obtained from the unsaturated images of harmonic spectra. The installation of gold-coated plane mirror (PM) or gold-coated cylindrical mirror inside the XUV spectrometer allowed for analyzing the divergence of harmonics or collecting the focused images of harmonics, respectively. The gold-coated mirror significantly improves the reflectivity of XUV radiation at the 3^0^ blaze angle of interaction; and it does not oxidize.

The raw images of harmonics showed their variations in different spectral ranges using single-color and two-color field configurations at $I_{\omega }$ = 1.2 × 10^14^ W cm^−2^ and $I_{\omega }$ = 1 × 10^13^ W cm^−2^ intensities of the fundamental and SH pulses. Figure 1b shows the line-outs of harmonic spectra generated in N_2_ and O_2_ diatomic molecules using two-color field in the case of 1.5 mm thick BBO crystal. The stronger even harmonics at 2(2*n* + 1) orders (H14, H18, H22), where *n* is an integer number and H is harmonic order, were observed in these gases using orthogonally-polarized two-color laser pulses. The stronger CE of 2(2*n* + 1) harmonics is attributed to the imperfect temporal overlap between the orthogonally-polarized two-color pulses after propagating through the 1.5-mm thick BBO crystal and due to the stronger yield of harmonics from the shorter-wavelength source. Meanwhile, even the small temporal overlap between the driving pulses became sufficient for two-wave interaction in the gas jet.

The group velocity dispersion in BBO leads to a temporal separation of the two pulses. Due to the difference in group velocities in the type-I BBO crystal the 1030 nm pulse became delayed by δ*t* = *l*_cryst_[(*n*^*o*^_*ω*_)_group_*/c* − (*n*^*e*^_2*ω*_)_group_*/c*] ≈ 12 fs with respect to the 515 nm pulse due to *n*^*o*^_*ω*_ > *n*^*e*^_2*ω*_ in this negative uniaxial crystal. Correspondingly, the 0.4-mm thick crystal causes 48 fs delay between the two pulses. The pulse duration of the generated SH (Δ*t*_2*ω*_) can be estimated using the following Eq. ^42,43^:

4 $$ \Delta t_{2\omega } = \sqrt {\delta t^{2} + \frac{{\left( {\Delta t_{\omega } } \right)^{2} }}{2}} $$

Here $\Delta t_{\omega }$ is the duration of fundamental pulse and $\delta t$ is the temporal shift between ω and 2ω waves induced by the group velocity dispersion in the nonlinear crystal. Using the 37 fs main pulses and the calculated delay between two pulses, the SH pulse durations in the case of 0.1- and 0.4-mm thick crystals were calculated to be 29 and 54 fs, respectively.
